# SGLT5 Reabsorbs Fructose in the Kidney but Its Deficiency Paradoxically Exacerbates Hepatic Steatosis Induced by Fructose

**DOI:** 10.1371/journal.pone.0056681

**Published:** 2013-02-25

**Authors:** Taku Fukuzawa, Masanori Fukazawa, Otoya Ueda, Hideaki Shimada, Aki Kito, Mami Kakefuda, Yosuke Kawase, Naoko A. Wada, Chisato Goto, Naoshi Fukushima, Kou-ichi Jishage, Kiyofumi Honda, George L. King, Yoshiki Kawabe

**Affiliations:** 1 Research Division, Chugai Pharmaceutical Co., Ltd., Gotemba, Shizuoka, Japan; 2 Chugai Research Institute for Medical Science, Inc., Gotemba, Shizuoka, Japan; 3 Research Division, Joslin Diabetes Center, Harvard Medical School, Boston, Massachusetts, United States of America; The University of Manchester, United Kingdom

## Abstract

Although excessive fructose intake is epidemiologically linked with dyslipidemia, obesity, and diabetes, the mechanisms regulating plasma fructose are not well known. Cells transfected with sodium/glucose cotransporter 5 (SGLT5), which is expressed exclusively in the kidney, transport fructose in vitro; however, the physiological role of this transporter in fructose metabolism remains unclear. To determine whether SGLT5 functions as a fructose transporter in vivo, we established a line of mice lacking the gene encoding SGLT5. Sodium-dependent fructose uptake disappeared in renal brush border membrane vesicles from SGLT5-deficient mice, and the increased urinary fructose in SGLT5-deficient mice indicated that SGLT5 was the major fructose reabsorption transporter in the kidney. From this, we hypothesized that urinary fructose excretion induced by SGLT5 deficiency would ameliorate fructose-induced hepatic steatosis. To test this hypothesis we compared SGLT5-deficient mice with wild-type mice under conditions of long-term fructose consumption. Paradoxically, however, fructose-induced hepatic steatosis was exacerbated in the SGLT5-deficient mice, and the massive urinary fructose excretion was accompanied by reduced levels of plasma triglycerides and epididymal fat but fasting hyperinsulinemia compared with fructose-fed wild-type mice. There was no difference in food consumption, water intake, or plasma fructose between the two types of mice. No compensatory effect by other transporters reportedly involved in fructose uptake in the liver and kidney were indicated at the mRNA level. These surprising findings indicated a previously unrecognized link through SGLT5 between renal fructose reabsorption and hepatic lipid metabolism.

## Introduction

Epidemiological studies and nutritional experiments have shown that excessive consumption of fructose is closely linked with metabolic abnormalities including dyslipidemia, obesity, diabetes, and cardiovascular disease [1], [2], and the dramatic increase in consumption of fructose, which is used to sweeten a wide variety of foods and soft drinks, has been proposed as a possible causal factor [3], [4]. In animal experiments, it is also shown that high fructose intake induces these abnormalities [5], [6]. Fructose is mainly metabolized by the liver, where it is a highly lipogenic substrate [7]. High fructose concentration enhances hepatic *de novo* lipogenesis and plays a critical role in the pathogenesis of nonalcoholic fatty liver disease (NAFLD) and may promote the transition from NAFLD to a more severe pathophysiological phenotype: nonalcoholic steatohepatitis (NASH) [8]. Excess triglyceride accumulation causes insulin resistance by directly interfering with hepatic insulin signaling [9]; however, it is still unclear whether there is a causal relationship between hepatic steatosis and insulin resistance [10]. These fructose-induced metabolic abnormalities, which underlie type 2 diabetes and cardiovascular diseases, are the reason that fructose metabolism has recently begun to gain the attention of researchers.

Most monosaccharides, including fructose, glucose, galactose, and mannose, as well as *myo*-inositol are transported across the cell membrane by either of two types of glucose transporters: facilitated glucose transporters (GLUTs) and sodium/glucose cotransporters (SGLTs). Several reports suggest that fructose is transported across the intestinal epithelium by GLUT2 and GLUT5 [11], [12], [13], [14], [15], [16]. Especially, GLUT5 knockout mouse [17] showed a 75% decrease of fructose absorption in the jejunum and 90% decrease in serum fructose concentration, indicating the primary role of GLUT5 in intestinal fructose absorption. Fructose exists in a free form in the plasma and is rapidly absorbed into the liver by GLUT2 and to a lesser extent into other tissues through GLUT2, GLUT8, GLUT9, and GLUT12 [12], [18], [19], where it can then be utilized as an energy source. Increased flux of fructose into the liver significantly enhances the rate of *de novo* lipogenesis and triglyceride synthesis by up-regulating lipogenic enzymes [6].

Plasma fructose concentration is potentially regulated in the kidney also. This is considered likely because plasma glucose concentration is strictly regulated by renal glucose reabsorption in addition to the glucose utilization in tissues such as the liver, skeletal muscle, and adipose. SGLT1 and SGLT2 contribute to the regulation of renal glucose uptake, and the induction of urinary glucose excretion with SGLT2 inhibition has been shown to reduce plasma glucose in clinical trials with diabetic patients [20], [21]. Several glucose transporters, such as GLUT2, GLUT5, NaGLT1, and SGLT4, have been reported to transport fructose and exist in the kidney [15], [22], [23], [24]; however, the contribution of these transporters to renal fructose transport in the regulation of plasma fructose levels is not known. Additionally, it has been recently reported that human SGLT5 is exclusively expressed in the kidney and that, in *in vitro* experiments using cells overexpressing human SGLT5, it mediates the transport of fructose and mannose [25]; however, the actual physiological role of SGLT5 *in vivo* has remained unknown.

In the current study, we generated mice lacking *Slc5a10,* the gene encoding SGLT5. We first confirmed the activity of SGLT5 as a fructose transporter by using renal brush border membrane vesicles (BBMVs) of the mice, and we then identified the function of SGLT5 as a renal transporter that reabsorbs fructose. Since inhibition of renal glucose reabsorption by an SGLT2 inhibitor appears to be an attractive anti-diabetic strategy [20], we hypothesized that inhibition of renal fructose reabsorption would increase urinary excretion of fructose and thereby prevent fructose-induced metabolic abnormalities. We tested this hypothesis by using SGLT5-deficient mice under conditions of long-term high fructose consumption.

## Materials and Methods

### Animals

All mice were housed under a 12-h/12-h light/dark cycle (lights on 7:00 AM–7:00 PM) with controlled room temperature (20–26°C) and humidity (35–75%), and were allowed *ad libitum* access to standard mouse chow (CE-2;Clea Japan, Tokyo, Japan). All animal care and experiments were performed in accordance with the guidelines for the care and use of laboratory animals at Chugai Pharmaceutical Co., Ltd. The protocol was approved by the Institutional Animal Care and Use Committee at Chugai Pharmaceutical Co. Ltd. (Permit No. 11-237). All surgery was performed under isoflurane anesthesia, and all efforts were made to minimize suffering.

### Generation of SGLT5-deficient mice and genotyping

The *Slc5a10* knockout mouse was established basically by the same protocol as we described previously [26], [27], [28] with some modifications. The protocol is briefly described as follows. A clone of a bacterial artificial chromosome (BAC) carrying C57BL/6 (B6) mouse genomic DNA containing all exons of the *Slc5a10* gene was engineered by using the Red/ET system (Gene Bridges, Heidelberg, Germany) for construction of a *Slc5a10* gene-targeting vector [29], [30]. The targeting vector was introduced by electroporation into B6 mouse embryonic stem cells (ES cells). Homologous recombinant ES cell clones were injected into BALB/c mouse blastocysts to produce chimeric mice. Chimeric mice were bred with B6 females to establish *Slc5a10* gene-deficient mice, which were named B6-*Slc5a10^tm1Csk^*. Heterozygous mice were intercrossed to produce homozygous mice. Polymerase chain reaction (PCR) for genotyping was performed with a common forward primer Sg5-4401 (5′-AGTACACAGGGACTGTCAGGC-3′) and different reverse primers: SgR-5309 (5′- GGAGCTTCTTACGGATGTCT-3′) for wild-type and Neo-anti4 (5′- AACTTCCTGACTAGGGGAGGAGTA-3′) for null allele.

### Tissue distribution analysis of mouse SGLT5

Expressions of mouse SGLT5 (mSGLT5) were determined by using mouse 1st strand cDNA (GenoStaff, Tokyo, Japan). Quantitative RT-PCR was performed on an ABI 7000 Sequence Detection System (Applied Biosystems, Foster City, CA) using primers specific for SGLT5. Expression of GAPDH was used as an internal control.

### Uptake experiments in COS-7 cells

Expression plasmids containing human SGLT5 (hSGLT5) and mSGLT5 were prepared by the ligation of fragments, amplified from Human Kidney Marathon-Ready cDNA (Clontech Laboratories, Mountain View, CA) or cDNA fragments prepared from the kidney of db/db mouse with primers designed from published sequences (GenBank accession numbers: NM001042450, NM001033227), into the multi-cloning site of pcDNA3.1(−) (Life Technologies Corporation [Invitrogen], Grand Island, NY). The expression plasmids containing hSGLT5 or mSGLT5 cDNA fragments were transfected into African green monkey SV40-transfected kidney fibroblast cells (COS-7; ATCC), and the cells transiently expressing each SGLT were used for the fructose uptake assay. For fructose uptake assay, the cells expressing each SGLT were cultured in 96-well plates for 2 days and washed twice with sodium-free buffer (140 mM choline chloride, 2 mM KCl, 1 mM CaCl_2_, 1 mM MgCl_2_, 10 mM HEPES-Tris, pH 7.4). The cells were then incubated in the sodium-free buffer or in sodium buffer (140 mM NaCl, 2 mM KCl, 1 mM CaCl_2_, 1 mM MgCl_2_, 10 mM HEPES-Tris, pH 7.4) each containing 0.1 mM fructose (mixture of non-radiolabeled fructose and [^14^C]-fructose (Moravek Biochemicals, Brea, CA)) at 37°C for 40 min. Sodium-dependent fructose uptake was calculated by subtracting the radioactivity detected in cells incubated in the sodium-free buffer from the radioactivity detected in the cells incubated in the sodium buffer.

### Preparation of renal brush border membranes (BBMs)

Kidneys were collected from anesthetized mice and homogenized in ice-cold isolation buffer (50 mM mannitol, 2 mM Hepes-Tris, pH 7.1) with an Ace homogenizer (Model AM-1; Nissei, Tokyo, Japan) at 18,000 rpm for 2 min. CaCl_2_ (1 M) was added to give a final concentration of 10 mM. After stirring for 20 min at 4°C, the suspension was centrifuged at 3,000×*g* for 15 min at 4°C. The supernatant was then centrifuged at 39,800×*g* for 30 min at 4°C. The pellets were suspended in suspension buffer (100 mM mannitol, 10 mM Hepes-Tris, pH 7.5) and homogenized with a Teflon homogenizer on ice. The samples were centrifuged at 39,800×*g* for 30 min at 4°C. The final pellets containing the BBMs were suspended in suspension buffer and stored at −80°C until use. Determination of activity of membrane marker enzymes revealed enrichment of alkaline phosphatase (ALP) in the final membrane preparations.

### Renal brush border membrane vesicle (BBMV) assay

The protein concentration of the BBM suspensions was adjusted to 6.3 mg/mL with suspension buffer. The samples were then suspended by passing 10 times through a 22- and 27-gauge needle. Uptake reaction was initiated by adding 20 µL of membrane suspension to 20 µL potassium buffer (100 mM KCl, 50 mM mannitol, 5 mM Hepes-Tris, pH 7.5) or sodium buffer (100 mM NaCl, 50 mM mannitol, 5 mM Hepes-Tris, pH 7.5) containing either [^14^C]-d-fructose or [^14^C]-d-mannose (Moravek Biochemicals, Brea, CA). Mixed samples were incubated for 10 s at 25°C. Incubation was terminated by adding 1 mL ice-cold stop solution containing 100 mM fructose or mannnose, followed by filtration. The mixture was poured immediately onto nitrocellulose membrane filters (Millipore, Billerica, MA), and the filters were washed with 5 mL of ice-cold stop buffer. The radioactivity of the filter was counted in 10 mL Clear-sol I (Nacalai Tesque, Tokyo, Japan) by a Tricarb 3110TR scintillation counter (PerkinElmer, Boston, MA). Sodium-independent fructose or mannose uptake was calculated from the radioactivity detected in the sample incubated in the sodium-free buffer. Sodium-dependent fructose or mannose uptake was calculated by subtracting the radioactivity detected in the sample incubated in the sodium-free buffer from the radioactivity detected in the sample incubated in the sodium buffer.

### Long-term fructose consumption

Wild type (WT) C57BL/6 mice and SGLT5-deficient mice (*n* = 8–10 per group, 11-week-old male) had free access to 30% (wt/vol) fructose water or plain water for 11 weeks. Plasma glucose levels, body weight, food intake, and water intake were measured periodically. Plasma glucose levels were measured by a plasma glucose monitoring system (Accu-Chek Aviva; Roche Diagnostics, Tokyo, Japan). The mice were maintained in metabolic cages and 24-h urine samples were collected at 21 weeks of age. Then an oral glucose challenge (2 g/kg) was administered to the conscious mice after fasting for 16 h, and the plasma glucose levels were measured at 30, 60, 120, 180 min after glucose ingestion. At 22 weeks of age, plasma, liver, kidney, and epididymal fat were collected from each mouse under anesthesia with isoflurane.

### Hepatic triglyceride determination

Livers were homogenized in PBS/methanol (1∶2.5). The same volume of chloroform as PBS was added to the homogenate and mixed vigorously. The samples were centrifuged at 1,200×*g* for 10 min. The lower layer was collected, dried, and suspended in isopropyl alcohol. Hepatic triglyceride level was quantified with a Triglyceride E-test kit (Wako Pure Chemicals, Osaka, Japan).

### Histopathology

Collected livers were embedded in OCT compound (Sakura Finetek, Tokyo, Japan) in cryomolds and frozen in liquid nitrogen. Liver sections were cut and stained with Sudan III, hematoxylin-eosin (HE) and Sirius red by Sapporo General Pathology Laboratory Co., Ltd, Sapporo, Japan) and then examined under a light microscope.

### Biochemical analysis

Biochemical analysis for plasma total cholesterol, triglycerides, alanine aminotransferase (AST), aspartate aminotransferase (ALT), lactate dehydrogenase (LDH), alkaline phosphatase (ALP), creatine kinase, total bilirubin, urea nitrogen, creatinine, and albumin was done with an automated chemistry analyzer (TBA-120FR; Toshiba Medical Systems, Tokyo, Japan). Urinary and plasma fructose concentrations were measured with a commercially available kit (Enzytec fluid d-Glucose/d-Fructose; J.K. International, Tokyo, Japan). Plasma insulin levels were determined with an insulin ELISA kit (Morinaga Institute of Biological Science, Kanagawa, Japan).

### Expression analysis of transporters in the liver and kidney

Total RNA was isolated from the liver and kidney using an RNeasy Minikit (Qiagen, Valencia, CA) according to the manufacturer's instructions. Quantitative RT-PCR was performed on an ABI PRISM 7900 Sequence Detection System (Applied Biosystems) using the QuantiTect Probe RT-PCR Kit with probes specific for transcripts of each transporter or *MAPK1*. Relative gene expression was calculated with the 2^−ΔCt^ method using *MAPK1* as an endogenous control.

### Microarray experiments

From the livers of WT mice and SGLT5-deficient mice that had been given either plain water or fructose water we isolated total RNA by using an RNeasy Minikit (Qiagen, Valencia, CA) according to the manufacturer's instructions. RNA quality was determined by analysis on an Agilent 2100 Bioanalyzer (Agilent Technologies, Santa Clara, CA). For profiling gene expression in the liver, Mouse Genome 430 2.0 Arrays (Affymetrix, Santa Clara, CA) were hybridized with RNA isolated from livers in each of the four groups, washed, and scanned according to the standard Affymetrix protocol. Expression profiles were analyzed using Genespring GX 11.5.1 software (Agilent Technologies). After background correction, we performed normalization using GCRMA. Genes were filtered by intensity values (lower cut off percentile of 20% for raw signals) under each of the four combinations of: +/+ Plain water; −/−Plain water; +/+Fructose water; −/− Fructose water. Gene activity was considered to differ between conditions if P values were <0.05 when compared by one-way ANOVAs (with the false discovery rate (FDR) multiple testing error correction procedure of Benjamini and Hochberg) followed by Tukey's post hoc test (with the same P cut-off value). The array data has been submitted to the public microarray database, ArrayExpress repository (Accession No. E-MEXP-3734; http://www.ebi.ac.uk/arrayexpress)

### Statistical analysis

Statistical analysis was performed with the SAS System for Windows, Release 8.02 (SAS Institute Japan, Tokyo, Japan). Statistical significance was determined by Student's *t*-test unless otherwise specified. *P*<0.05 was regarded as statistically significant.

## Results

### SGLT5 expression in mouse kidney and fructose transport

**Figure 1:**
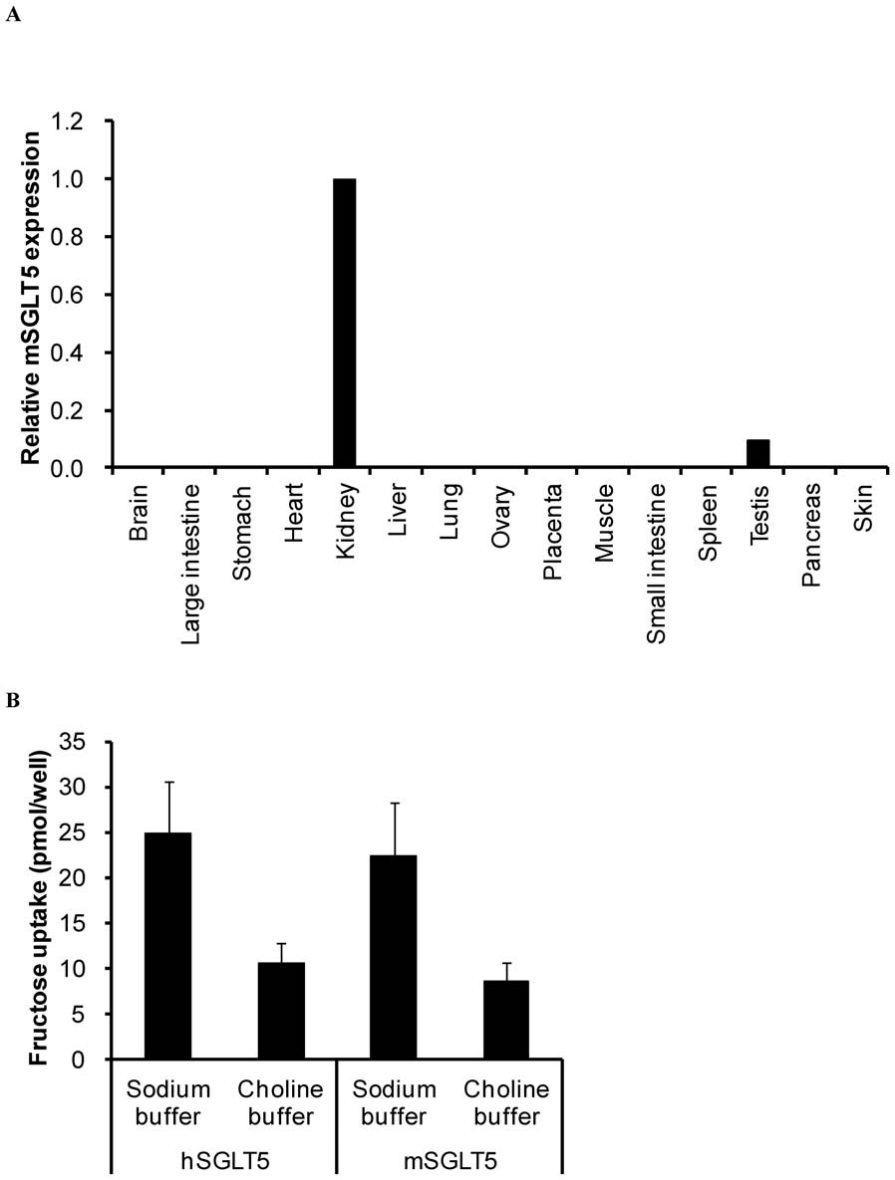
SGLT5 distribution and fructose uptake. (A) Tissue distribution of mouse SGLT5 and (B) SGLT5-mediated fructose uptake in COS-7 cells. Data are presented as means ± S.D. Data are derived from 3 independent experiments.

Firstly we confirmed mSGLT5 tissue expression in mice by quantitative RT-PCR on a panel of mouse tissue samples. As shown in Figure 1A, mSGLT5 was almost exclusively expressed in the kidney; only slight expression of mSGLT5 was observed in the testis, and no amplification product was obtained in the remaining 13 tissues. This expression pattern was similar to that of hSGLT5 [25]. To investigate the ability of SGLT5 to facilitate uptake of fructose, we transiently transfected hSGLT5 or mSGLT5 into COS-7 cells and performed uptake assays with ^14^C-labeled fructose. We observed that both human and mouse SGLT5 transported fructose in a sodium-dependent manner (Figure 1B).

### Generation of SGLT5-deficient mice

**Figure 2:**
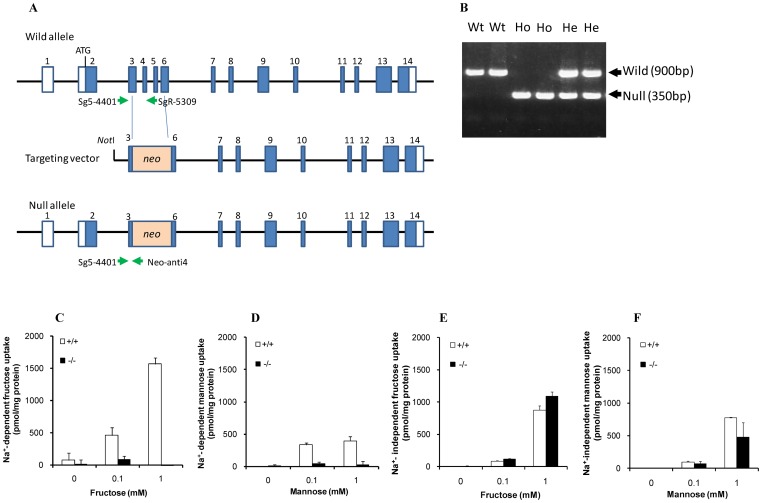
Generation of SGLT5-deficient mice and their fructose and mannose uptake by renal BBMV s. (A) Schematic representation of the strategy for targeting the *Slc5a10* gene. A targeting vector was constructed by inserting a neomycin resistant (*neo*) gene cassette to disrupt exons 3–6 of the *Slc5a10* genomic locus on a BAC genomic clone. Arrows indicate PCR primers for genotyping. (B) A representative result of genotyping the offspring obtained by intercrossing heterozygous-deficient mice. Wild type and null alleles are detected as signals of 900 bp and 350 bp, respectively. *Wt*: Wild type mice, *He*: Heterozygous null mutant, *Ho*: Homozygous null mutant. (C) Sodium-dependent uptake of fructose and (D) mannose in BBMVs of WT mice (+/+) and SGLT5-deficient mice (−/−). (E) Sodium-independent uptake of fructose and (F) mannose in BBMVs of WT mice (+/+) and SGLT5-deficient mice (−/−). Data are presented as means ± S.D. Data are derived from 3 independent experiments.

We generated SGLT5-deficient mice by disruption of the *Slc5a10* gene in ES cells by using standard homologous recombination techniques (Figure 2A). A representative result of PCR analysis for genotyping is shown in Figure 2B. Homozygous null mice were obtained in the ratio expected by Mendelian inheritance and have no apparent morphological abnormalities.

### Fructose and mannose uptake by renal BBMV from SGLT5-deficient mice

To confirm that SGLT5 transports fructose in renal tubules, we assayed renal BBMVs prepared from the kidneys of WT or SGLT5-deficient mice. Figure 2C shows sodium-dependent uptake of fructose in BBMVs. In the presence of 0.1 mM and 1 mM fructose, the fructose uptake observed in BBMVs from WT mice was absent in BBMVs from SGLT5-deficient mice. The sodium-independent uptake of fructose was observed with lesser activity compared to sodium-dependent uptake, and no significant differences in the sodium-independent fructose uptake between WT and SGLT5-decficient mice (Figure 2E). Mannose uptake was also observed in BBMVs from WT mice, but the activity was lower than that observed for fructose uptake (Figure 2D). The sodium-dependent mannose uptake was also observed in BBMVs from WT mice, but the activity was lower than that observed for fructose uptake and this mannose transport activity in BBMVs from mice lacking SGLT5 was lower than that in normal BBMVs (Figure 2D). The sodium-independent uptake of mannose was not significantly different between WT and SGLT5-decficient mice (Figure 2F). The sodium-dependent glucose and galactose uptake were not affected by SGLT5 deficiency (data not shown). These results clearly show that mSGLT5 functions mainly as a fructose and also as a mannose transporter in the kidney.

### Urinary fructose reabsorption in SGLT5-deficient mice

**Figure 3:**
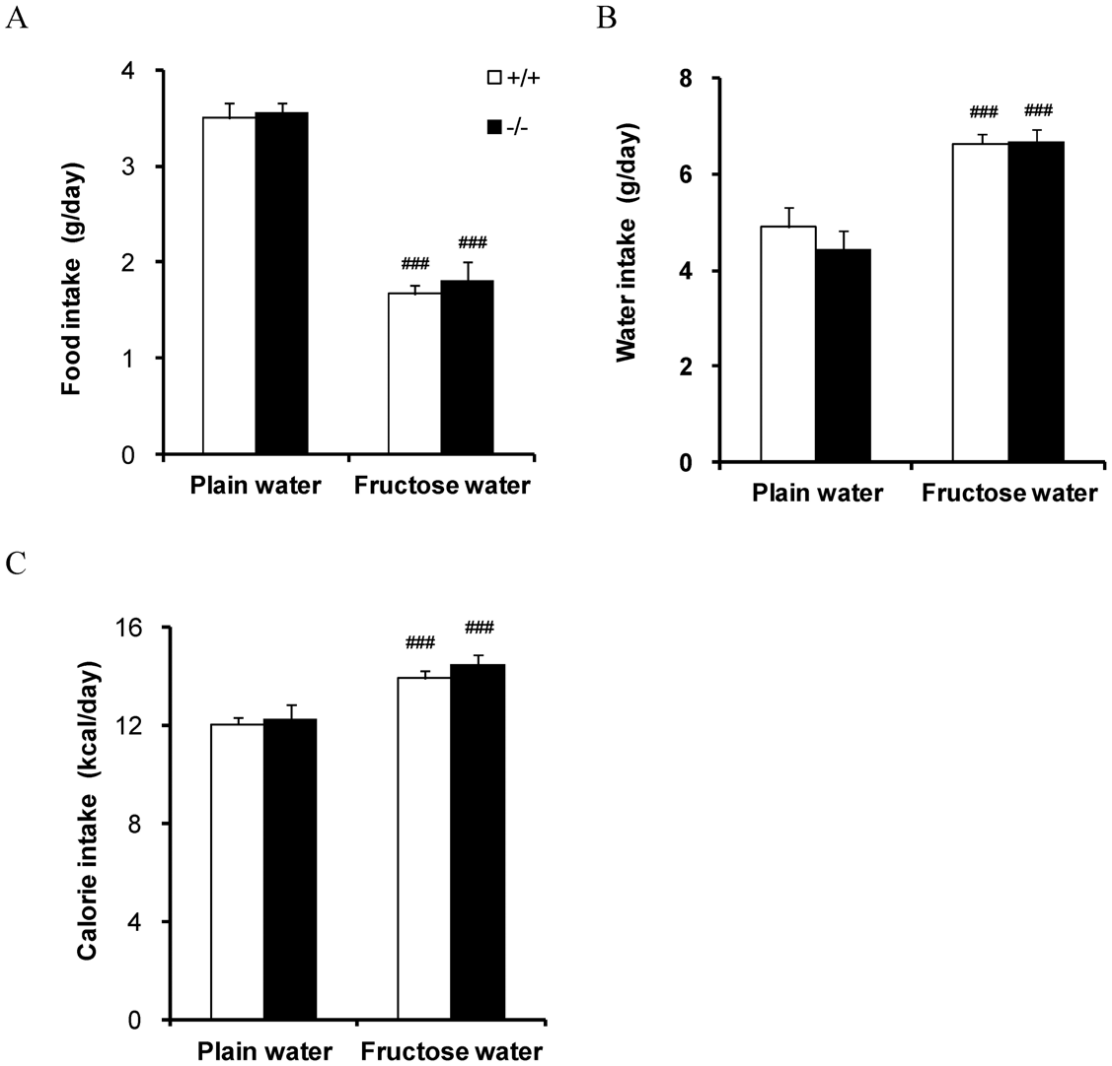
Food and water intake in WT (+/+) mice and SGLT5-deficient mice (−/−). Daily intake of (**A**) **food and** (**B**) **water of mice at 17 weeks of age.** (C) Calculated daily energy intake. Data are presented as means ± S.E.M (n = 8–10). ### P<0.001 versus respective plain water control.

**Figure 4:**
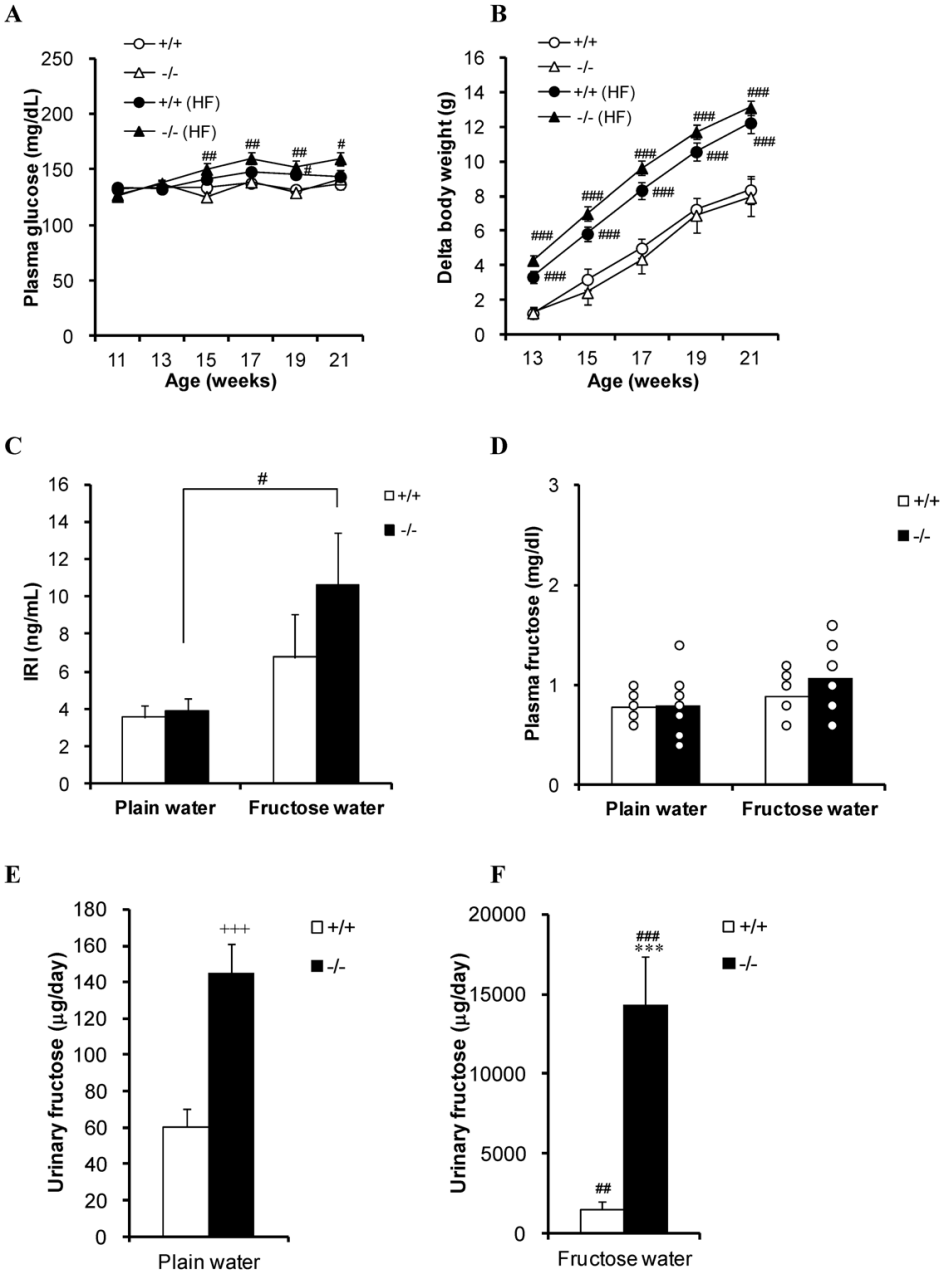
Effect of high fructose consumption in WT mice and SGLT5-deficient mice. (A) Plasma glucose levels of WT mice (+/+) and SGLT5-deficient mice (−/−) were measured every 2 weeks. *HF*: Mice given water containing high fructose. (B) Growth curves of WT mice and SGLT5-deficient mice. (C) Plasma samples were collected after 6 h fasting at 21 weeks of age, and immunoreactive insulin (*IRI*) was determined. (D) Plasma fructose concentrations measured in plasma samples collected under anesthesia after 3 h fasting. *Open circles* represent individual data. (E and F) WT mice and SGLT5-deficient mice given plain water or fructose water were maintained in metabolic cages and 24-h urine samples were collected. Urinary fructose excretion was calculated by multiplying urinary fructose concentration by the amount of urine. Data are presented as means ± S.E.M (*n* = 8–10). *** *P*<0.001 versus WT mice given 30% fructose water. # *P*<0.05, ## *P*<0.01, ### *P*<0.001 versus respective plain water controls. +++ *P*<0.001 versus WT mice given plain water.

Since sodium-dependent fructose transport was impaired in BBMVs of SGLT5-deficient mice, we investigated whether SGLT5 affected fructose metabolism *in vivo*. WT and SGLT5-deficient mice (*n* = 8–10, 11 weeks old) had free access to 30% fructose water or plain water for 11 weeks. Although fructose consumption reduced food intake and raised water intake, and slightly increased total energy intake per day, no differences in food or water consumption were observed between WT and SGLT5-deficient mice (Figure 3A, B, C). WT and SGLT5-deficient mice given 30% fructose water respectively received 58.7% and 56.7% of their daily energy intake as fructose. During the experimental period, fructose intake increased plasma glucose levels and body weights, but there was no significant difference between WT and SGLT5-deficient mice (Figure 4A, B). Blood insulin level tended to increase in WT mice and SGLT5-deficient mice given fructose, and in SGLT5-deficient mice this increase in insulin was significant (Figure 4C).

Urine and plasma samples were collected at 21 and 22 weeks of age, respectively. As shown in Figure 4D, there was no significant difference in plasma fructose levels between groups. In mice given plain water, urinary fructose excretion was extremely low but was obviously higher in SGLT5-deficient mice than in WT mice (Figure 4E). When given high fructose water, urinary fructose excretion was elevated in both WT and SGLT5-deficient mice. The amount of urinary fructose of SGLT5-deficient mice was considerably higher than that of normal mice (Figure 4F). These results clearly show that there is a defect in the renal fructose reabsorption system in SGLT5-deficient mice, which leads to massive urinary fructose excretion.

### Long-term effect of fructose consumption in SGLT5-deficient mice

Excess fructose intake leads to increased hepatic triglyceride levels in animals [31], [32]. Since an excessive amount of fructose was excreted by SGLT5-deficient mice as compared to WT mice, we examined the hypothesis that inhibition of renal fructose reabsorption would accelerate fructose disposition and thereby prevent fructose-induced metabolic abnormalities in these mice.

**Figure 5:**
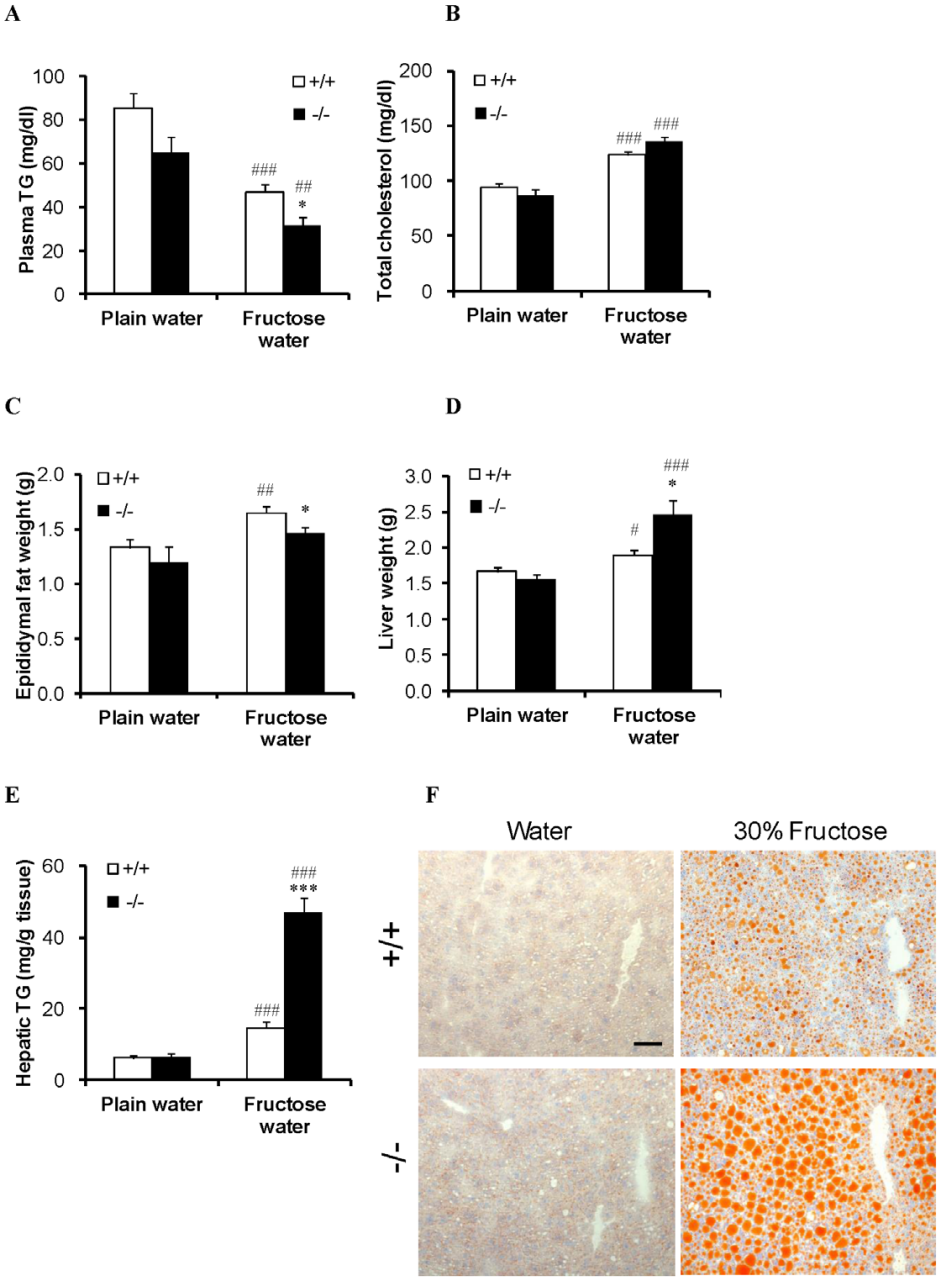
Influence of the long-term consumption of fructose on tissue weight and lipid metabolism. (A) Plasma triglyceride levels of WT mice (+/+) and SGLT5-deficient mice (−/−). (B) Plasma total cholesterol levels. (C) Weight of epididymal fat. (D) Weight of the liver. (E) Hepatic triglyceride levels. (F) Histopathological analysis of the liver sections. Two sections per mouse were stained with Sudan III. Representative images are shown (scale bar, 50 µm). Data are presented as means ± S.E.M (*n* = 8–10). * *P*<0.05, *** *P*<0.001 versus WT mice given 30% fructose water. # *P*<0.05, ## *P*<0.01, ### *P*<0.001 versus respective plain water controls.

In WT mice, high fructose intake decreased plasma triglyceride level (Figure 5A) but increased plasma total cholesterol level (Figure 5B), epididymal fat amount (Figure 5C), liver weight (Figure 5D), and hepatic triglyceride level (Figure 5E). In SGLT5-deficient mice, levels of plasma triglycerides and epididymal fat were found to be lower than those in WT mice, both with plain water consumption and with fructose water consumption (Figure 5A, C). But plasma total cholesterol concentration in SGLT5-deficient mice was not different from that in WT mice under either of these conditions (Figure 5B). To our surprise, however, triglycerides were massively accumulated in the liver of SGLT5-deficient mice receiving 30% fructose water (Figure 5E). The elevated hepatic triglyceride content was associated with increased liver weight and severe hepatic steatosis (Figure 5D, F) as shown by the increased ratio of mice with severe accumulation of lipid droplets in the liver (Table S1, Figure S1). No obvious inflammatory or fibrogenic changes were detected in the HE or Sirius red-stained liver sections of the SGLT5-deficient mice receiving 30% fructose water, except for increased vacuolation (data not shown), which may be due to the increased accumulation of lipid droplets.

**Figure 6:**
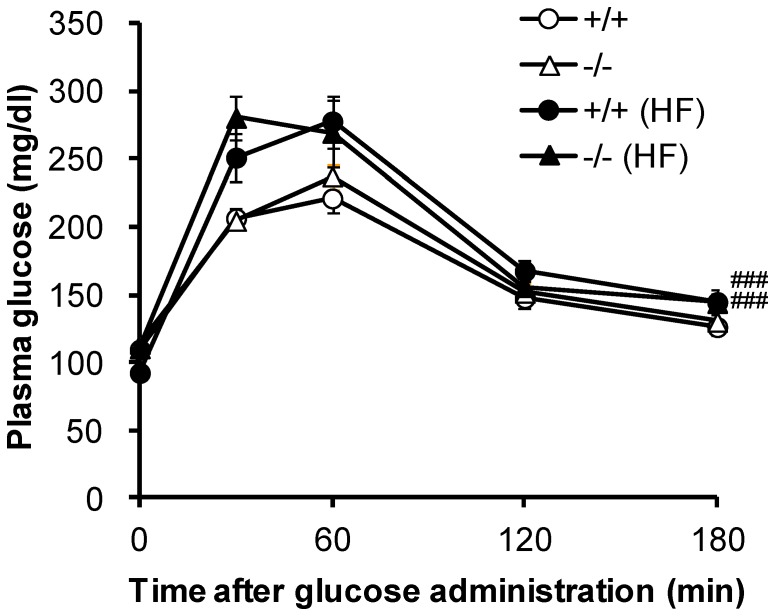
Oral glucose tolerance test with WT mice (+/+) and SGLT5-deficient mice (−/−) given plain water or fructose water (HF). Fasted 21-week-old male mice received an oral dose of glucose (2 g/kg). Plasma glucose levels were determined at the indicated time points. Data are presented as means ± S.E.M (*n* = 8–10). ### *P*<0.001 versus respective water controls by analysis of covariance (ANCOVA).

An oral glucose tolerance test revealed that both the WT and the SGLT5-deficient mice given water containing fructose exhibited glucose intolerance, but no significant difference between WT and SGLT5-deficient mice was observed (Figure 6). The severe fatty liver observed in SGLT5-deficient mice was also associated with increased plasma ALT and AST, which are commonly used parameters reflecting hepatic impairment, including NAFLD (Table 1) [33]. Additionally, LDH, a tissue injury marker, was increased in SGLT5-deficient mice given fructose water (Table 1) [34].

**Table 1:** Characteristics of 22-week-old WT mice (+/+) and SGLT5-deficient mice (−/−) receiving plain water or fructose water (HF).

	+/+	−/−	+/+ (HF)	−/− (HF)
AST (U/L)	33.7±0.8	43.4±8.1	39.9±1.3###	58±7.1*
ALT (U/L)	24.4±1.9	27.1±4.2	29.7±1.8	63.3±13.4* #
ALP (U/L)	186.3±4.9	196.9±14.2	259.9±7.0###	291.6±23.0##
LDH (U/L)	172.1±8.1	184.1±19.0	211.3±10.6##	293±30.9* #
Creatine kinase (U/L)	58.6±5.3	69.3±5.3	111.4±54.9	109.6±21.8
Total bilirubin (mg/dl)	0.079±0.004	0.1±0.029	0.082±0.005	0.089±0.006
BUN (mg/dl)	21.7±1.0	19.81±0.062	14.0±0.7###	15.3±0.7###
Creatinine (mg/dl)	0.061±0.002	0.064±0.003	0.067±0.003	0.073±0.012
Albumin (g/dl)	2.75±0.048	2.76±0.043	3.07±0.021###	3.2±0.069* ###

Data are presented as means ± S.E.M (n = 8–10).

* P<0.05 versus WT mice given 30% fructose water.

# P<0.05,

## P<0.01,

### P<0.001 versus respective plain water controls. AST: aspartate aminotransferase, ALT: alanine aminotransferase, ALP: alkaline phosphatase, LDH: lactate dehydrogenase, BUN: blood urea nitrogen.

### Gene expression analysis

**Figure 7:**
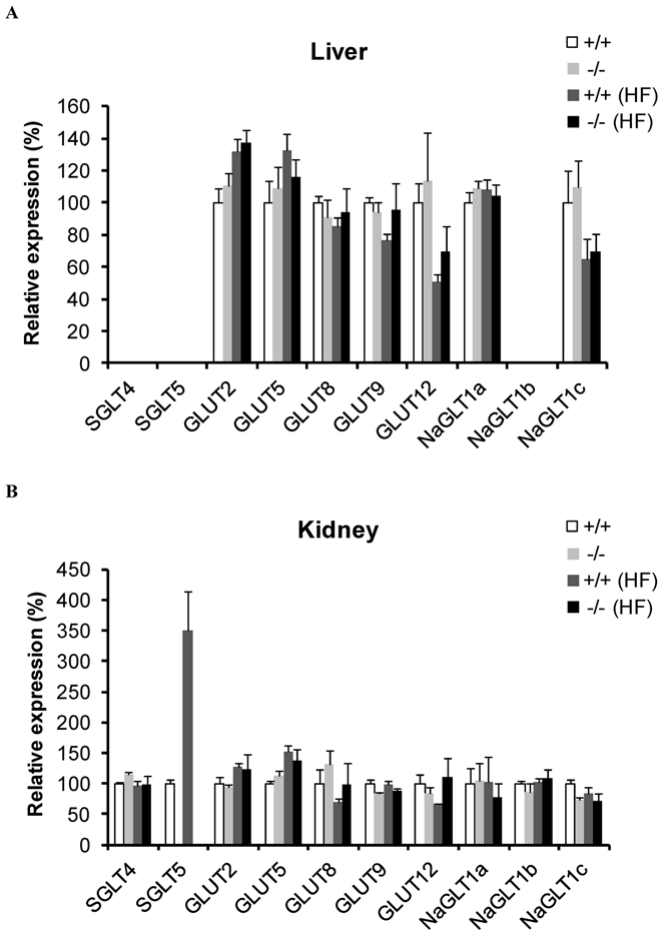
Gene expression analysis. Quantitative RT-PCR was performed with total RNA isolated from (A) the liver and (B) the kidney of WT mice (+/+) and SGLT5-deficient mice (−/−) given plain water or high fructose (HF) water. *MAPK1* was used as an internal control. Data are presented as means ± S.E.M. *n* = 3 (A). *n* = 8–10 (B).

Excessive accumulation of hepatic triglycerides in SGLT5-deficient mice led us firstly to examine the possibilities that the lack of fructose uptake via SGLT5 induces up-regulation of other fructose transporters in the liver or compensation in the kidney. We therefore performed quantitative RT-PCR to determine expression levels of transporters possibly involved in fructose uptake in the liver and kidney; these included SGLT4, SGLT5, GLUT2, GLUT5, GLUT8, GLUT9, GLUT12, NaGLT1a, NaGLT1b, and NaGLT1c. In the liver, there was no significant difference between groups in the expression levels of the transporters, suggesting that hepatic fructose absorption might be normal in SGLT5-deficient mice (Figure 7A). In the kidney, SGLT5 was up-regulated in WT mice receiving fructose. However, there was no change in the expression levels of the nine transporters other than SGLT5 between fructose-fed WT mice and SGLT5-deficient mice (Figure 7B). These results precluded the possibility that any of these nine other transporters examined here contributed to the exaggeration of hepatic triglyceride accumulation in SGLT5-deficient mice.

Next, to obtain insight into the molecular mechanisms underlying the exacerbation of hepatic steatosis induced by fructose in the SGLT5-deficient mice, we used microarray analysis to compare the liver gene expression patterns of the four groups (Figure S2). We first examined the effect of fructose consumption on gene expression in WT mice. From among the 45,101 probe sets on the microarray, fructose consumption increased expression of 86 probe sets (fold change of 1.5 or more) with respect to average expression in WT mice given plain water. Of these, probe sets for the leptin receptor and ApoA4 genes were those most induced (fold change of approx. 10). On the other hand, we observed 103 down-regulated probe sets (fold change of 1.5 or more) with fructose consumption (data not shown). We then examined the effect of SGLT5 deletion on gene expression in mice, but only minimal changes, and no changes in lipogenic factors including target genes of SREBP-1 and gluconeogenic and glycolytic enzymes were observed (Table S2). Interestingly, the probe set for Cyp17a1 was increased by fructose consumption in WT mice (fold change of 3.17) and further increased in SGLT5-deficient mice (fold change of 4.46); however, to our knowledge, there is no report indicating a relationship between triglyceride metabolism and the enzyme encoded by this gene.

## Discussion

In the present study, we identified the function of SGLT5 as a renal fructose reabsorption transporter by showing that sodium-dependent fructose uptake was diminished in renal BBMVs from SGLT5-deficient mice and that urinary fructose was increased in SGLT5-deficient mice. SGLT5 deficiency increased urinary fructose excretion without affecting plasma fructose concentrations and reduced plasma triglyceride levels. Unexpectedly, however, SGLT5 deficiency paradoxically exacerbated hepatic steatosis induced by long-term fructose consumption. The current results showed that inhibition of renal fructose reabsorption did not prevent fructose-induced metabolic abnormalities by increasing urinary fructose excretion. Instead, an intriguing link through SGLT5 between fructose reabsorption and hepatic lipid metabolism is suggested.

It is known that GLUT5, a fructose transporter, is present on the apical membrane of renal proximal tubules [35] in addition to being in the intestine, where it plays a critical role in fructose absorption [13], [14], [15], [17]. NaGLT1 is also known to be involved in fructose absorption in rat kidneys [23]. Both GLUT5 and NaGLT1, however, are low-affinity fructose transporters with respective *K*_m_ values for fructose of 6 mM and 7.8 mM [12], [23]. Under normal conditions, physiological blood fructose concentrations are much lower than those values (under 0.05 mM) both in humans [36], [37] and in our WT mice, suggesting the existence of a high-affinity fructose transporter. Although Horiba *et al.* reported high-affinity fructose transport activity (*K*_m_ value of 0.3 mM) in rat kidneys [23], the molecular entity of that transporter remains unknown. In this study, we observed sodium-dependent uptake of fructose at 0.1 mM via SGLT5 in COS-7 cells and in wild-type mice BBMVs, which lose their fructose uptake ability in SGLT5-deficient mice. These findings, together with the other data in this study, suggest that SGLT5 is a high-affinity fructose transporter that potentially functions in the kidneys of humans and rodents.

GLUT5 is expressed only in the S3 segment of renal proximal tubules [35]. Because the basolateral fructose transporter GLUT2 is localized only in S1 and S2 [38], transporters other than GLUT5 can also be working on the apical side of these segments. In situ hybridization shows that SGLT2, which exists in segments S1 and S2 [39], and SGLT5 are co-expressed in epithelial tissue of the glomerulus and distal and proximal tubules [40]. Although we have no direct evidence showing the presence of this transporter in proximal tubules because a specific SGLT5 antibody is not available, we looked at fructose uptake in a BBMV assay. This assay method had been validated by testing the effect of several SGLT2 inhibitors on sodium-dependent glucose transport (data not shown) and, in the case of sodium-dependent fructose transport, detected it only in wild-type mice. Taking this result together with the results of the present *in vivo* study, in which SGLT5 deficiency increased urinary fructose excretion, we suggest that SGLT5, a high-affinity fructose transporter, incorporates fructose at the apical membrane of the proximal tubule in collaboration with GLUT2 and contributes primarily to renal fructose reabsorption.

Since accelerated urinary excretion of fructose was demonstrated in SGLT5-deficient mice, we used these mice under conditions of high fructose consumption to test the hypothesis that inhibition of renal fructose reabsorption would prevent fructose-induced metabolic abnormalities. SGLT5 deficiency obviously elevated fructose excretion. Nonetheless, compared with fructose-fed WT mice, fructose-fed SGLT5-deficient mice exhibited more severe hepatic steatosis, which was associated with raised levels of inflammatory and tissue damage markers including AST and ALT. Since the fructose intake of WT mice and SGLT5-deficient mice was equivalent, and SGLT5 deficiency facilitated fructose excretion, the abnormal lipid accumulation observed in the liver of SGLT5-deficient mice is not accounted for by a quantitative balance between intake and excretion of fructose. These findings indicate that fructose-induced metabolic abnormalities are not ameliorated by inhibiting renal fructose reabsorption through SGLT5 deficiency, and that there may be unique features involved in SGLT5 deficiency.

To investigate the cause of exacerbated hepatic steatosis in SGLT5-deficient mice, we compared the gene expression levels of possible fructose transporters in the kidney and in the liver. However, there was no significant difference in expression levels between WT and SGLT5-deficient mice, suggesting that the cause of exacerbated hepatic steatosis is not likely to be excess fructose uptake in these tissues. Nevertheless, we cannot exclude the possibility of increases in the protein levels of other fructose transporters or increases in the translocation of GLUTs to the cell surface. *De novo* extra-hepatic lipogenesis, such as adipogenesis, is probably not the major causative factor of the increased level of hepatic triglycerides in SGLT5-deficient mice because consumption of fructose-containing water resulted in decreased levels of plasma triglycerides and epididymal fat compared to levels in fructose-fed WT mice. Although decreased hepatic lipoprotein secretion is a possible explanation, this is not supported by the similar plasma total cholesterol levels in the SGLT5-deficient and WT mice consuming fructose.

From these results and the discussion above, we speculated that hepatic *de novo* lipogenesis is further enhanced by SGLT5 deletion. Although our liver gene expression profiling using a microarray did not support this speculation, other mechanisms such as post-transcriptional regulation (e.g., processing of SREBP-1c precursor to the active nuclear form) might be a possible mechanism. This and previous studies [25] indicate that SGLT5 also transports mannose, suggesting that impaired processes of glycosylation involving mannose is another possibility.

Increased insulin resistance is implicated by the finding that fasting insulin and glucose intolerance increased in SGLT5-deficient mice under fructose consumption. Hepatic insulin resistance is also suggested by the lack of change in the liver gene expression profile of lipogenic factors, including target genes of SREBP-1, gluconeogenic enzymes, and glycolytic enzymes; however, this finding also suggests lipid accumulation per se is not regulated at transcriptional level. The complexity of insulin action, which uses a variety of mechanisms, including post-transcriptional regulation, and is itself modulated both by allosteric regulation of responsible enzymes and by the availability of substrates for lipogenesis and gluconeogenesis, means that further study will be needed to understand the causal relationship between hepatic steatosis insulin resistance and fructose metabolism in the liver and kidney.

Although details of the mechanisms underlying the increased levels of hepatic triglycerides in SGLT5-deficient mice remain unclear for now, our finding has important implications with respect to a previously unknown link between the renal fructose reabsorption system and hepatic lipid metabolism. Further investigations elucidating the molecular mechanisms underlying this link would provide a new therapeutic option with which to combat metabolic disorders such as hepatic steatosis, dyslipidemia, obesity, and diabetes.

## Supporting Information

Figure S1 **Representative figures of the liver sections from WT mice and SGLT5-deficient mice receiving plain water or fructose water. ±, +, ++, +++: see grade of lipid droplets in Table S1.** Staining: Sudan III (scale bar: 50 µm).
(PDF)

Figure S2 **Two-way hierarchical clustering analysis of 12 samples (4 conditions) and 469 genes.** Of the 45,101 genes represented in the microarray, 469 genes were altered between conditions (P<0.05 with a false discovery rate). The fold change ratio was calculated with respect to the average intensity of Condition 1. Purple is up-regulated and blue is down-regulated with respect to the average intensity of Condition 1; black indicates no change. +/+, WT mice; −/−, SGLT5-deficient mice.
(PDF)

Table S1 (PDF)

Table S2 (PDF)
