# Supplementary material for: SGLT5 Reabsorbs Fructose in the Kidney but Its Deficiency Paradoxically Exacerbates Hepatic Steatosis Induced by Fructose
Source: PLoS One. 2013 Feb 25;8(2):e56681. doi: 10.1371/journal.pone.0056681 (PMC3581502; doi:10.1371/journal.pone.0056681)
Supplement: Figure S1 — Representative figures of the liver sections from WT mice and SGLT5-deficient mice receiving plain water or fructose water. ±, +, ++, +++: see grade of lipid droplets in Table S1. Staining: Sudan III (scale bar: 50 µm). (PDF) [file pone.0056681.s001.pdf]

Figure S1

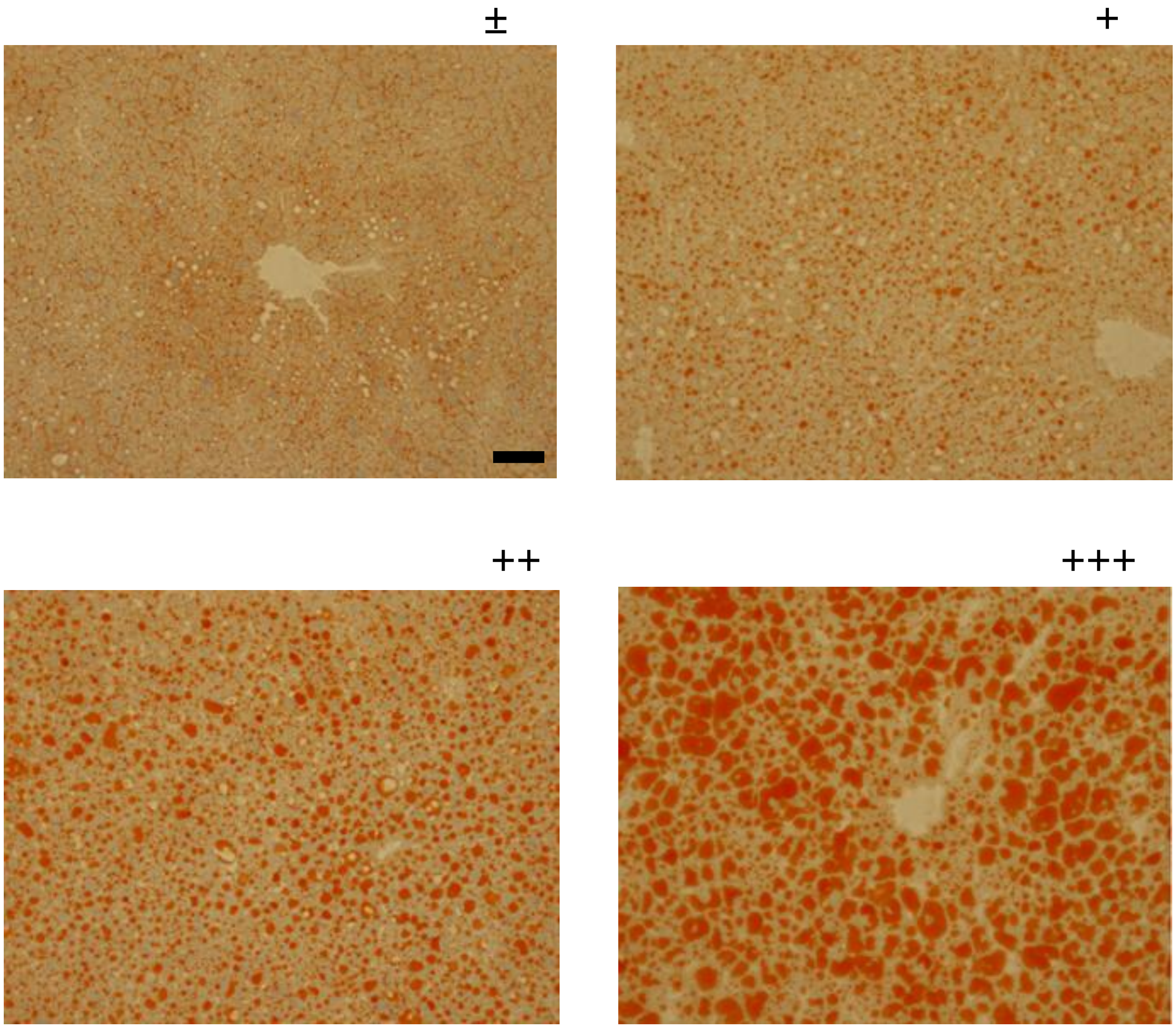

Figure S1. Representative figures of the liver sections from WT mice and SGLT5-deficient mice receiving plain water or fructose water. ±, +, ++, +++: see grade of lipid droplets in **Table S1**. Staining: Sudan III (scale bar: 50 μm).
