# Supplementary material for: SGLT5 Reabsorbs Fructose in the Kidney but Its Deficiency Paradoxically Exacerbates Hepatic Steatosis Induced by Fructose
Source: PLoS One. 2013 Feb 25;8(2):e56681. doi: 10.1371/journal.pone.0056681 (PMC3581502; doi:10.1371/journal.pone.0056681)
Supplement: Figure S2 — Two-way hierarchical clustering analysis of 12 samples (4 conditions) and 469 genes. Of the 45,101 genes represented in the microarray, 469 genes were altered between conditions (P<0.05 with a false discovery rate). The fold change ratio was calculated with respect to the average intensity of Condition 1. Purple is up-regulated and blue is down-regulated with respect to the average intensity of Condition 1; black indicates no change. +/+, WT mice; −/−, SGLT5-deficient mice. (PDF) [file pone.0056681.s002.pdf]

Figure S2.

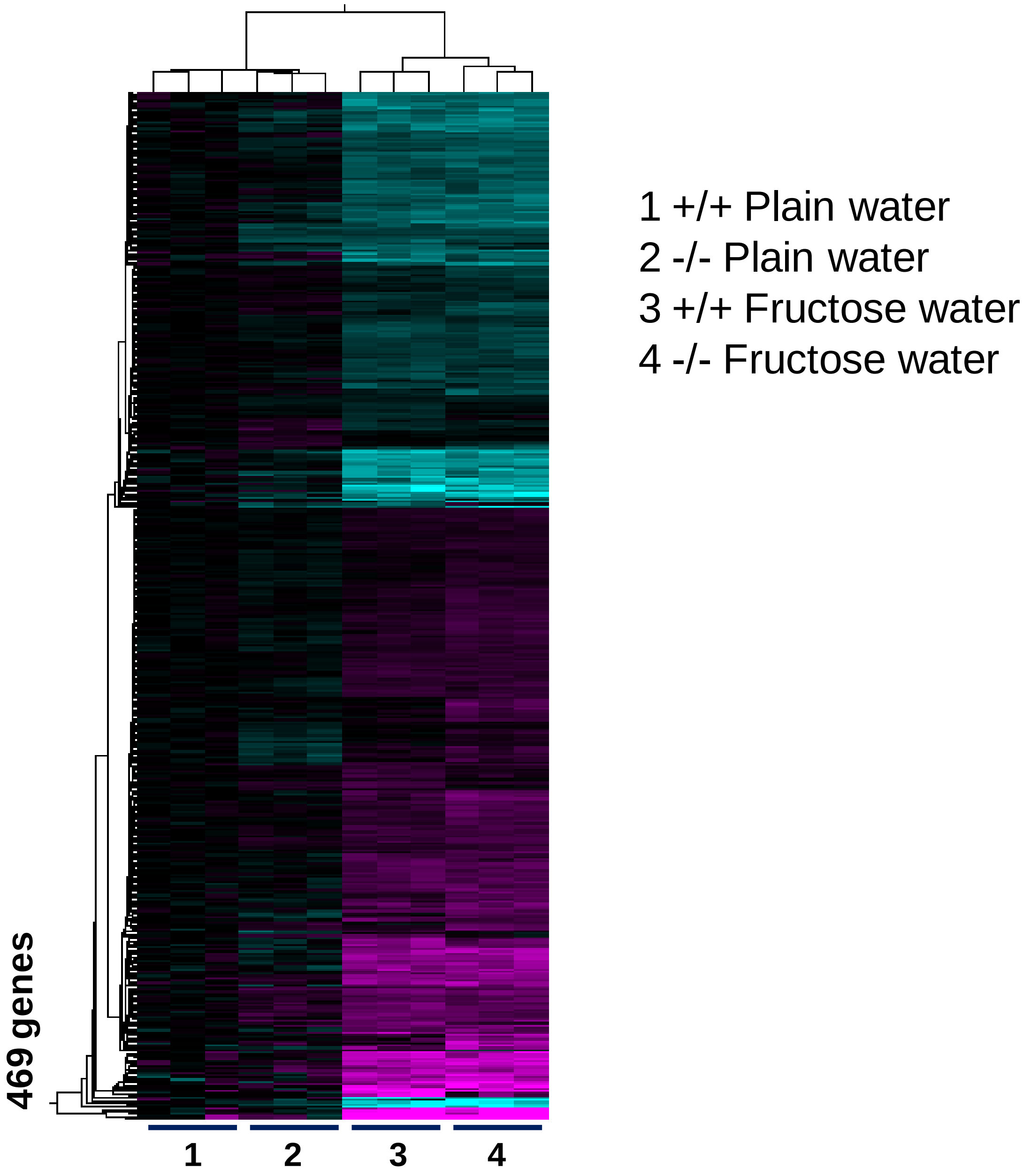

Figure S2. Two-way hierarchical clustering analysis of 12 samples (4 conditions) and 469 genes. Of the 45,101 genes represented in the microarray, 469 genes were altered between conditions ($P < 0.05$ with a false discovery rate). The fold change ratio was calculated with respect to the average intensity of Condition 1. Purple is up-regulated and blue is down-regulated with respect to the average intensity of Condition 1; black indicates no change. +/+, WT mice; −/−, SGLT5-deficient mice.
