# Supplementary material for: SGLT5 Reabsorbs Fructose in the Kidney but Its Deficiency Paradoxically Exacerbates Hepatic Steatosis Induced by Fructose
Source: PLoS One. 2013 Feb 25;8(2):e56681. doi: 10.1371/journal.pone.0056681 (PMC3581502; doi:10.1371/journal.pone.0056681)
Supplement: Table S1 — (PDF) [file pone.0056681.s003.pdf]

Table S1. Grading of lipid droplets in the liver of WT mice (+/+) and SGLT5-deficient mice (-/-) receiving plain water or fructose water (HF)

Genotype	n	Number of mice<br>Grade of lipid droplets				
		–	±	+	++	+++
+/+	10	1	8	1	0	0
-/-	10	0	10	0	0	0
+/+ (HF)	10	0	0	4	6	0
-/- (HF)	9	0	0	2	3	4

–: no droplet, ±: fine droplets (very slight), +: small droplets (slight), ++: middle droplets (moderate), +++: large droplets (severe). The representative figures for each grade are shown in **Figure S1**.
