# Supplementary material for: SGLT5 Reabsorbs Fructose in the Kidney but Its Deficiency Paradoxically Exacerbates Hepatic Steatosis Induced by Fructose
Source: PLoS One. 2013 Feb 25;8(2):e56681. doi: 10.1371/journal.pone.0056681 (PMC3581502; doi:10.1371/journal.pone.0056681)
Supplement: Table S2 — (PDF) [file pone.0056681.s004.pdf]

**Table S2.** List of differentially expressed genes in livers of SGLT5 knockout mice (FDR ≤ 0.05, fold change ≥ 1.5).

| **Genes down-regulated by SGLT5 deficiency (Plain water)** | | | | |
|---|---|---|---|---|
| Probe Set ID | Fold change | Gene Symbol | Entrez Gene | Gene Title |
| 1433691_at | -1.74 | Ppp1r3c | 53412 | protein phosphatase 1, regulatory (inhibitor) subunit 3C |
| 1427408_a_at | -1.66 | Thrap3 | 230753 | thyroid hormone receptor associated protein 3 |
| 1456489_at | -1.60 | Pcf11 | 74737 | cleavage and polyadenylation factor subunit homolog (S. cerevisiae) |
| 1423905_at | -1.54 | Pvr | 52118 | poliovirus receptor |
| 1435193_at | -1.50 | A230050P20Rik | 319278 | RIKEN cDNA A230050P20 gene |
| 1423904_a_at | -1.50 | Pvr | 52118 | poliovirus receptor |

| **Genes up-regulated by SGLT5 deficiency (Plain water)** | | | | |
|---|---|---|---|---|
| Probe Set ID | Fold change | Gene Symbol | Entrez Gene | Gene Title |
| 1455260_at | 1.56 | Lcorl | 209707 | ligand dependent nuclear receptor corepressor-like |

| **Genes down-regulated by SGLT5 deficiency (Fructose water)** | | | | |
|---|---|---|---|---|
| Probe Set ID | Fold change | Gene Symbol | Entrez Gene | Gene Title |
| 1431406_at | -3.07 | Agxt2l1 | 71760 | alanine-glyoxylate aminotransferase 2-like 1 |
| 1433888_at | -2.84 | Atp2b2 | 11941 | ATPase, Ca++ transporting, plasma membrane 2 |
| 1439764_s_at | -2.50 | Igf2bp2 | 319765 | insulin-like growth factor 2 mRNA binding protein 2 |
| 1433691_at | -2.20 | Ppp1r3c | 53412 | protein phosphatase 1, regulatory (inhibitor) subunit 3C |
| 1438992_x_at | -2.07 | Atf4 | 11911 | activating transcription factor 4 |
| 1434436_at | -1.98 | Morc4 | 75746 | microrchidia 4 |
| 1427488_a_at | -1.62 | Birc6 | 12211 | baculoviral IAP repeat-containing 6 |
| 1452708_a_at | -1.60 | Luc7l | 66978 | Luc7 homolog (S. cerevisiae)-like |
| 1428170_at | -1.57 | Zfp180 | 210135 | zinc finger protein 180 |
| 1437667_a_at | -1.57 | Bach2 | 12014 | BTB and CNC homology 2 |
| 1438442_at | -1.57 | 5730470L24Rik | 66641 | RIKEN cDNA 5730470L24 gene |
| 1417374_at | -1.55 | Tuba4a | 22145 | tubulin, alpha 4A |
| 1435926_at | -1.53 | Chml | 12663 | choroideremia-like |
| 1416418_at | -1.50 | Gabarapl1 | 57436 | gamma-aminobutyric acid (GABA) A receptor-associated protein-like 1 |

| **Genes up-regulated by SGLT5 deficiency (Fructose water)** | | | | |
|---|---|---|---|---|
| Probe Set ID | Fold change | Gene Symbol | Entrez Gene | Gene Title |
| 1417017_at | 4.46 | Cyp17a1 | 13074 | cytochrome P450, family 17, subfamily a, polypeptide 1 |
| 1453416_at | 2.60 | Gas2l3 | 237436 | growth arrest-specific 2 like 3 |
| 1437813_at | 2.17 | Aim1l | 230806 | absent in melanoma 1-like |
| 1416191_at | 2.09 | Sec61a1 | 53421 | Sec61 alpha 1 subunit (S. cerevisiae) |
| 1460591_at | 1.98 | Esr1 | 13982 | estrogen receptor 1 (alpha) |
| 1424794_at | 1.88 | Rnf186 | 66825 | ring finger protein 186 |
| 1442612_at | 1.79 | C730036E19Rik | 402734 | RIKEN cDNA C730036E19 gene |
| 1435171_at | 1.79 | 2810416G20Rik | 100040353 | RIKEN cDNA 2810416G20 gene |
| 1434510_at | 1.76 | Papss2 | 23972 | 3'-phosphoadenosine 5'-phosphosulfate synthase 2 |
| 1444846_at | 1.64 | D15Ertd529e | 52510 | DNA segment, Chr 15, ERATO Doi 529, expressed |
| 1424613_at | 1.64 | Gprc5b | 64297 | G protein-coupled receptor, family C, group 5, member B |
| 1448978_at | 1.61 | Ngef | 53972 | neuronal guanine nucleotide exchange factor |
| 1418911_s_at | 1.59 | Acsl4 | 50790 | acyl-CoA synthetase long-chain family member 4 |
| 1455320_at | 1.57 | | | |
| 1417936_at | 1.57 | Ccl9 | 20308 | chemokine (C-C motif) ligand 9 |
| 1421989_s_at | 1.54 | Papss2 | 23972 | 3'-phosphoadenosine 5'-phosphosulfate synthase 2 |
| 1435043_at | 1.53 | Plcb1 | 18795 | phospholipase C, beta 1 |
| 1418219_at | 1.51 | Il15 | 16168 | interleukin 15 |
